# Hyaluronan, Cancer-Associated Fibroblasts and the Tumor Microenvironment in Malignant Progression

**DOI:** 10.3389/fcell.2018.00048

**Published:** 2018-05-08

**Authors:** James B. McCarthy, Dorraya El-Ashry, Eva A. Turley

**Affiliations:** ^1^Department of Laboratory Medicine and Pathology, Masonic Comprehensive Cancer Center, Minneapolis, MN, United States; ^2^London Regional Cancer Program, Department of Oncology, Biochemistry and Surgery, Schulich School of Medicine and Dentistry, Lawson Health Research Institute, Western University, London, ON, Canada

**Keywords:** hyaluronan, cancer-associated fibroblasts, migration, tumor microenvironment, tumor initiation, circulating cancer-associated fibroblasts, metastasis

## Abstract

This review summarizes the roles of CAFs in forming a “cancerized” fibrotic stroma favorable to tumor initiation and dissemination, in particular highlighting the functions of the extracellular matrix component hyaluronan (HA) in these processes. The structural complexity of the tumor and its host microenvironment is now well appreciated to be an important contributing factor to malignant progression and resistance-to-therapy. There are multiple components of this complexity, which include an extensive remodeling of the extracellular matrix (ECM) and associated biomechanical changes in tumor stroma. Tumor stroma is often fibrotic and rich in fibrillar type I collagen and hyaluronan (HA). Cancer-associated fibroblasts (CAFs) are a major source of this fibrotic ECM. CAFs organize collagen fibrils and these biomechanical alterations provide highways for invading carcinoma cells either under the guidance of CAFs or following their epithelial to mesenchymal transition (EMT). The increased HA metabolism of a tumor microenvironment instructs carcinoma initiation and dissemination by performing multiple functions. The key effects of HA reviewed here are its role in activating CAFs in pre-malignant and malignant stroma, and facilitating invasion by promoting motility of both CAFs and tumor cells, thus facilitating their invasion. Circulating CAFs (cCAFs) also form heterotypic clusters with circulating tumor cells (CTC), which are considered to be pre-cursors of metastatic colonies. cCAFs are likely required for extravasation of tumors cells and to form a metastatic niche suitable for new tumor colony growth. Therapeutic interventions designed to target both HA and CAFs in order to limit tumor spread and increase response to current therapies are discussed.

## Introduction

Historically, cancers have been studied as diseases whose initiation and progression are caused by the mutation of key oncogenic “driver” genes, loss of suppressor genes and increasing mutational load resulting in genomic instability, immortalization, unrestrained growth and acquisition of colonizing potential (Hanahan and Weinberg, 2011; Garraway and Lander, 2013; Tomasetti et al., 2013; Vogelstein et al., 2013). More recent studies predict this concept of cancer initiation and progression is incomplete. Most genetic changes that are hallmarks of epithelial cancer are already present in pre-malignant lesions that rarely progress to frank cancer. For example, ultra-deep sequencing of 74 cancer genes in small biopsies of normal aged and sun-exposed human skin reveal a high mutation burden in most key drivers of cutaneous squamous cell carcinoma (Martincorena et al., 2015). These were estimated to be present in over a quarter of the keratinocytes in an epidermis that maintained its normal tissue architecture and physiological functions. A similar paradigm has been observed in other tissues. Endometriosis is a benign inflammatory lesion that is cancer-like in its local invasion and resistance to apoptosis but rarely transforms. Exome sequencing shows that over a quarter of these benign lesions harbor oncogenic driver gene mutations confined to the epithelial compartment that do not result in tumors (Anglesio et al., 2017). These clinical findings are remarkably consistent with experimental studies showing that the tumor phenotype is plastic. Tumor cells can be reverted into a normal growth state while retaining a highly mutated genome by blocking signaling pathways commonly activated by tumor microenvironment (Illmensee and Mintz, 1976; Hall et al., 1995; Wang et al., 2002; Kenny and Bissell, 2003; Postovit et al., 2008; Bizzarri et al., 2011; Northey et al., 2017).

Clues as to the factors required for a mutant genome to either manifest as a transformed phenotype or be restrained into apparent normalcy were initially provided by pioneering studies. The classic studies of B. Mintz brought initial attention to the plasticity of the mutant tumor phenotype and the key role of microenvironments in maintaining transformation (Illmensee and Mintz, 1976). Teratocarcinoma cells, injected into blastocysts, unexpectedly participated in normal tissue development rather than forming tumors. In another key report, chick embryos injected with an oncogenic virus only developed tumors at wound sites even though the viral genome was expressed in unwounded tissues (Dolberg et al., 1985). These original results predicted that while oncogenic insults (e.g., mutations, oncogenic viruses) are a first step toward initiation of cancer, the status of host microenvironment is critical and rate-limiting for disease initiation and progression. These predictions have fueled a synergistic interest in characterizing the properties of “cancerized” host tissue that collaborate with mutant epithelial cells to produce tumors, and drive progression and metastasis, as well as targeting these properties with novel therapeutics designed to manage this aspect of the disease (Radisky et al., 2007; Karn et al., 2015; Werb and Lu, 2015; Luo et al., 2016; Turley et al., 2016; Bridelance et al., 2017; Ghosh et al., 2017; Hutchenreuther and Leask, 2017; Zhan et al., 2017).

Host stroma is a complex mixture of phenotypically heterogeneous endothelial cells, pericytes, immune cells and fibroblasts. Normally, each of these cell types are required for tissue homeostasis, and contribute to the maintenance of tissue architecture and physiologically appropriate tissue functions. The collective paracrine signaling networks that sustain these functions have highly effective tumor-suppressor activity. Gene expression analyses have shown that the stroma surrounding tumors is altered from normal stroma, has lost its tumor suppressing activity and participates in rather than limits tumor initiation, growth and spread (Campisi, 1998; Dumont and Arteaga, 2002; Barsky and Karlin, 2006; Coppé et al., 2010; Bissell and Hines, 2011; Hinds and Pietruska, 2017). Expression differences in normal vs. cancer stroma have been mined to identify signatures that add independent prognostic information to classical epithelial biomarkers (Berdiel-Acer et al., 2014; Bedognetti et al., 2015; Nannini et al., 2015; Winslow et al., 2015; Colangelo et al., 2017; Petitprez et al., 2017). These unbiased analyses together with experimental evidence predict the critical importance of neovascularization, inflammation, immune tolerance and fibroblast activation in creating a “cancerized” microenvironment. In this review, we focus upon the roles of carcinoma-associated fibroblasts (CAFs), also known as tumor-associated fibroblasts (TAFs), in creating a remodeling extracellular matrix that drives tumor initiation and mediates tumor cell spread. We concentrate on the tissue polysaccharide, hyaluronan (HA), as a key contributing ECM component in stromal fibrosis and tumor progression. We conclude by reviewing current experimental interventions targeting both stroma ECM and/or CAF functions that may ultimately limit tumor spread and improve current therapies.

## Stromal extracellular matrix in carcinoma initiation and progression

It is now well-accepted that carcinomas behave like wounds, which force the host tumor microenvironment into a constant state of fibrotic repair (Dvorak, 1986). As with wound repair, carcinoma-associated stromal tissues undergo dynamic changes in cellular composition and extensive remodeling of extracellular matrix (ECM) as they progress. A particular feature of stromal ECM in cancers particularly pancreatic, prostate, lung and esophageal is its highly fibrotic structure that significantly impacts on progression, metastasis and response-to-therapy (Keely, 2011; Tung et al., 2015; Werb and Lu, 2015; Jiang et al., 2017). Although less well-studied, evidence suggests that chronic inflammation and pro-fibrotic changes in host stroma precede and instruct primary tumor initiation or formation of metastatic colonies by creating a microenvironment or niche favorable for transformation and growth. As examples, in healthy individuals with BRCA1 mutations that are at risk for breast cancer, stromal fibroblasts exhibit a CAF-like activation state (Etzold et al., 2016). Similarly healthy individuals with Li Fraumeni syndrome who bear germ line mutations in TP53 and are at an elevated risk of cancer exhibit “cancerization” of their stromal tissues (Pantziarka, 2015). In a mouse model of colon tumor initiation, both a chronically inflamed and fibrotic stroma are an essential pre-requisite for tumor initiation (Sasaki et al., 2014; Tanabe et al., 2016). There has therefore been an intense effort to understand the dynamic changes in stromal ECM composition to identify the changes that impact on cancer progression, metastasis and resistance to therapies.

## Biomechanical properties of tumor-associated stroma

**Figure 1:**
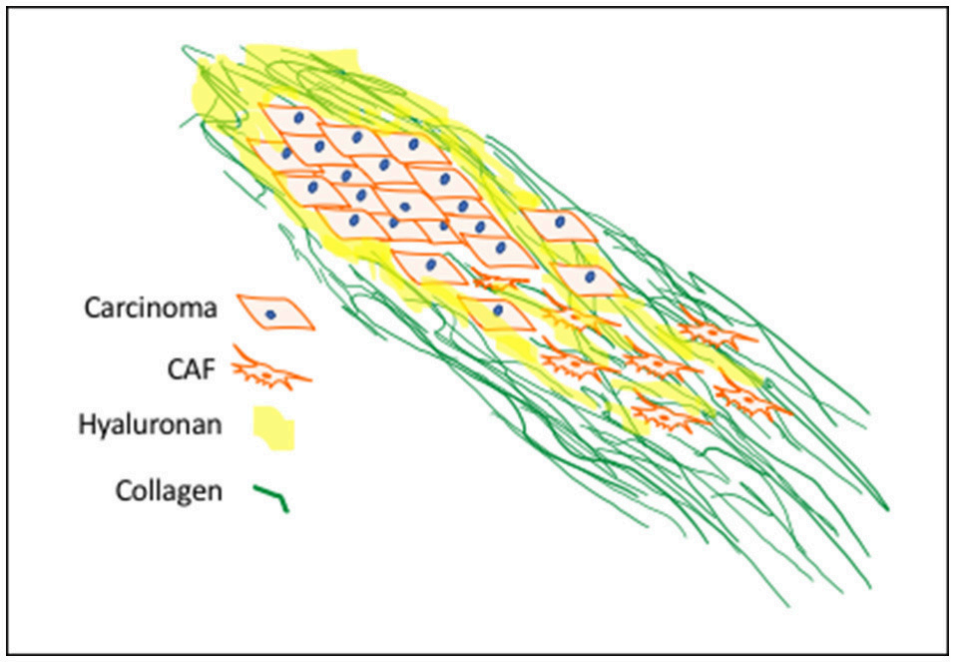
Progression-Associated Fibrosis in Cancerized Stroma: Deregulated synthesis and deposition of ECM components, including HA and type I collagen, leads to tumor-associated fibrosis. HA, a major polysaccharide of provisional wound matrices, contributes to cancer initiation, progression and resistance-to-therapy. CAF activation sustains increased collagen synthesis, structurally oriented by fibroblast contractile forces. These provide structural and biochemical cues to enhance mechano-signaling for carcinoma motility and invasion.

A major ECM component of all fibrotic stroma is type I collagen, which provides structural and biochemical cues to cells within the stroma (Keely, 2011; Tung et al., 2015; Werb and Lu, 2015; Jiang et al., 2017) (Figure 1). A notable property of “cancerized” stroma is the accumulation of type I collagen fibrils in the stroma that are extensively crosslinked by lysyl oxidase (LOX) and tissue transglutaminase (TG2) (Perryman and Erler, 2014; Lee et al., 2016). Collagen crosslinking confers proteolytic resistance to the fibrils and increases stroma stiffness, which promotes tumor cell migration, invasion and proliferation. Tumor-associated collagen signatures categorized by increased collagen density and orientation of mature collagen fibers parallel to or perpendicular to the tumor boundary offer prognostic information (Mellone et al., 2016). Orientation of the fibrils is the result of a process of prolonged mechano-signaling mediated by integrin/cytoskeletal linkages, activation of downstream adhesion pathway signaling components particularly focal adhesion kinase (FAK), phosphorylation of myosin light chain kinase and activation of Rho-Kinase (Schedin and Keely, 2011; Boyle and Samuel, 2016). Oriented collagen fibrils are an ominous biomarker of tumor cell invasion, metastasis and poor outcome (Schedin and Keely, 2011; Tung et al., 2015). In experimental models, non-transformed epithelial cell adhesion to stiff collagen matrices results in elevated activation of oncogenic pathways and increased expression of growth-promoting genes, emphasizing that the mechanical property of stiffness contributes to carcinoma progression (Paszek et al., 2005; Provenzano and Keely, 2011; Ray et al., 2017). Carcinoma cells cultured on stiff collagen gels grow as colonies with discrete boundaries, whereas the same cells cultured in oriented collagen gels of equal stiffness invade along these collagen fibers (Provenzano and Keely, 2011). These *in vitro* observations have been confirmed *in vivo* using multiphoton laser scanning microscopy and second harmonic generation imaging of live *ex-vivo* tumors (Provenzano and Keely, 2011). While fibrillar collagen is a major component of fibrotic stroma, many additional prognostic ECM factors impact the biological and biomechanical properties of tumor-associated stroma. One of these is HA, whose elevated accumulation in the tumor microenvironment contributes to cancer initiation, progression and therapy resistance (Karousou et al., 2014; Chanmee et al., 2016; Sato et al., 2016; Turley et al., 2016; Binder et al., 2017; Bourguignon et al., 2017; Safdar et al., 2017). These properties as they relate to tumor initiation and dissemination are discussed in the following sections below.

## Cancer-associated fibroblasts

Cancer-associated fibroblasts (CAF) are the primary cell type in “cancerized” stroma and are a major source of ECM as well as cytokines/growth factors that impact upon both tumor susceptibility/initiation and progression (Kalluri, 2016; Liu et al., 2017; Santi et al., 2017; Yamauchi et al., 2018). CAFs are a heterogeneous mixture of multiple resident fibroblast subtypes and infiltrated circulating mesenchymal cells. Understanding the origin and nature of the fibroblasts that drive oncogenic initiation and progression has been hampered by a paucity of CAF-specific markers and thus their origin remains controversial. Mesenchymal stem cells (MSCs) and resident fibroblast progenitors of CAFs are recruited by chemokines/cytokines and growth factors to specific sites and ECM components at these sites activate these cells into CAFs (Mishra et al., 2008; Shinagawa et al., 2010; Mi et al., 2011). For example, knockdown of the HA receptor CD44 in MSCs blocks both their ability to be recruited to the tumor site, and their tumor promoting functions (Spaeth et al., 2013) Recent studies have identified CAF properties that are distinct from activation of normal fibroblasts responding-to-wounding. For example, CAFs activation status appears to be irreversible while wound repair fibroblasts activation is both reversible and dependent on wound-induced signaling. The secretome, ECM remodeling and tumor promoting properties of CAFs and injury-activated fibroblasts also differ (Kalluri, 2016).

CAFs are most commonly identified by their expression of fibroblast activation protein (FAP) and alpha smooth muscle actin (∂-SMA), however, additional markers including platelet derived growth factor receptor b (PDGFRB), fibroblast specific protein (FSP) and vimentin (VIM), all of whose expression in tumor stroma have, like ∂-SMA, been linked to poor outcome of many cancers, can also be expressed in CAFs (Jacob et al., 2012; Folgueira et al., 2013; Paulsson and Micke, 2014; Han et al., 2015; Peiris-Pagès et al., 2015; Corvigno et al., 2016; Gascard and Tlsty, 2016; Kuzet and Gaggioli, 2016; Hammer et al., 2017; Tao et al., 2017; von Ahrens et al., 2017). The roles of CAFs as promoters of tumor initiation, progression, epithelial to mesenchymal transition, stemness, tumor invasion, angiogenesis, metastasis and drug resistance are well established (Kalluri and Zeisberg, 2006; Shekhar et al., 2007; Straussman et al., 2012) Experimentally, CAFs exhibit activity in all Hallmarks of Cancer categories (Salo et al., 2014; Tommelein et al., 2015; Attieh and Vignjevic, 2016; Mezawa and Orimo, 2016). Many studies of CAF participation in tumorigenesis have viewed their role as a reactive process that is a consequence of signals originating in the epithelial tumor, which results in a permissive environment for tumor cells to grow. A number of studies have demonstrated a more instructive role for CAFs in the initiation and dissemination of tumors. These studies have stimulated interest in the development of therapies that target CAFs and other stromal components of the tumor stroma. These CAF properties are reviewed here.

## CAFs and tumor initiation

In general, fibroblasts in normal stroma have tumor-suppressing properties (Bhowmick et al., 2004; Augsten, 2014; Klein, 2014; Rhee et al., 2015; Kubo et al., 2016; Lin and Lin, 2017; Mangge et al., 2017). However, when normal fibroblasts are activated (e.g., into myofibroblasts) or become senescent they lose these tumor-suppressing functions and under appropriate conditions convert into tumor-promoting and/or initiating CAFs. Experimentally, such cells can facilitate conversion of pre-malignant epithelial cells into tumors. An early example of this was provided by evidence that irradiated fibroblasts increase the incidence of tumors arising from pre-malignant mammary epithelial cells (Bhowmick et al., 2004; Ji et al., 2017). A number of more recent studies using experimental models provide direct evidence for the ability of CAFs to drive the initiation of cancer (Sasaki et al., 2014). Thus, loss or reduction of a notch effector (CSL) in stromal fibroblasts is sufficient for CAF activation and induction of keratinocyte tumors. Conversely, CCR5 blockade of fibroblast activation in colon tissue of a mouse model of colitis-associated carcinogenesis strongly reduces tumor initiation even though inflammation/colitis is still present. In experimental models, senescent fibroblasts have also been shown to enhance cancers including ovarian and keratinocyte transformation (Lawrenson et al., 2010).

Clinically, CAF-like fibroblast-induced stromal ECM changes have been reported to precede tumor formation and these early changes in ECM provide prognostic information that permit risk stratification. For example, high mammographic density is a strong risk factor in breast cancer (DeFilippis et al., 2012; Ghosh et al., 2017; Vinnicombe, 2017). Clinical features of this condition, which precede detectable tumor formation, include adipocyte loss and high ECM production. This condition has been linked to expression loss of the mesenchymal differentiation regulator CD36 in stromal fibroblasts, which phenocopies the clinical features of high mammographic density breast tissue. In clinical samples, CAFs exhibit loss of CD36 expression. (DeFilippis et al., 2012) and this in breast cancer tissue is strongly associated with poor outcome. Other examples include evidence that primary dermal fibroblasts exhibit a CAF-like state with a germ-line BRCA1 epi-mutation (Etzold et al., 2016). These fibroblasts stimulate rather than suppress epithelial proliferation and migration, express CAF markers including ACTA2, FAP, PDPN, and TNC, and are highly proliferative and migratory relative to normal counterparts from other patients. In early stage breast cancer, high stromal Heat Shock Factor 1 (HSF1) activation is associated with poor outcome and experimental data show that HSF1 expression is elevated/activated and results in potent enabling of malignancy (Scherz-Shouval et al., 2014). Genetic loci have been also identified that affect stromal properties and control mammary tumor susceptibility. These include genes that affect TGFß signaling (Zhang P. et al., 2015). Consistent with these findings, fibroblast-specific deletion of TGFßIIR in a transgenic mouse model results in repression of tumor suppressing functions of fibroblasts and a rapid development of aggressive prostate cancer (Li et al., 2012). HA is one ECM factor that is regulated by TGFß (Heldin et al., 2014) that is linked to tumor susceptibility, initiation and progression of many cancers and will be focused upon here.

## Stromal hyaluronan is linked to tumor susceptibility and CAF activation

HA is a simple extracellular matrix polysaccharide that a wealth of experimental approaches has demonstrated is an instructive factor in cancer initiation and progression (Heldin et al., 2014; Tolg et al., 2014; Zhang C. et al., 2015; Chanmee et al., 2016; Turley et al., 2016; Bohaumilitzky et al., 2017; Senbanjo and Chellaiah, 2017; Shih et al., 2017; Wight, 2017; Wong et al., 2017). For example, blocking HA synthesis (Itano et al., 2008; Hamada et al., 2017; Ikuta et al., 2017) or ablating the HA-binding function of one of its receptors RHAMM (gene name HMMR) (Hall et al., 1995), which has been strongly linked to tumorigenesis (Tolg et al., 2014; Turley et al., 2016), attenuates the transformed phenotype. Clinical analyses show that elevated HA accumulation in either the stroma or tumor parenchyma of many cancers is linked to tumor aggression and poor outcome (Sironen et al., 2011; McAtee et al., 2014; Chanmee et al., 2016; Sato et al., 2016; Turley et al., 2016; Bourguignon et al., 2017; Wu et al., 2017). Unexpectedly, HA has also recently been implicated as a stromal tumor-suppressing factor (Tian et al., 2013; Fisher, 2015; Triggs-Raine and Natowicz, 2015; Bohaumilitzky et al., 2017). These opposing effects are not well-understood but have been linked to differences in its metabolism and in particular the regulation of HA polymer size (Simpson and Lokeshwar, 2008; Tian et al., 2013; Khaldoyanidi et al., 2014; Tolg et al., 2014; Litwiniuk et al., 2016; Turley et al., 2016; Fouladi-Nashta et al., 2017).

**Figure 2:**
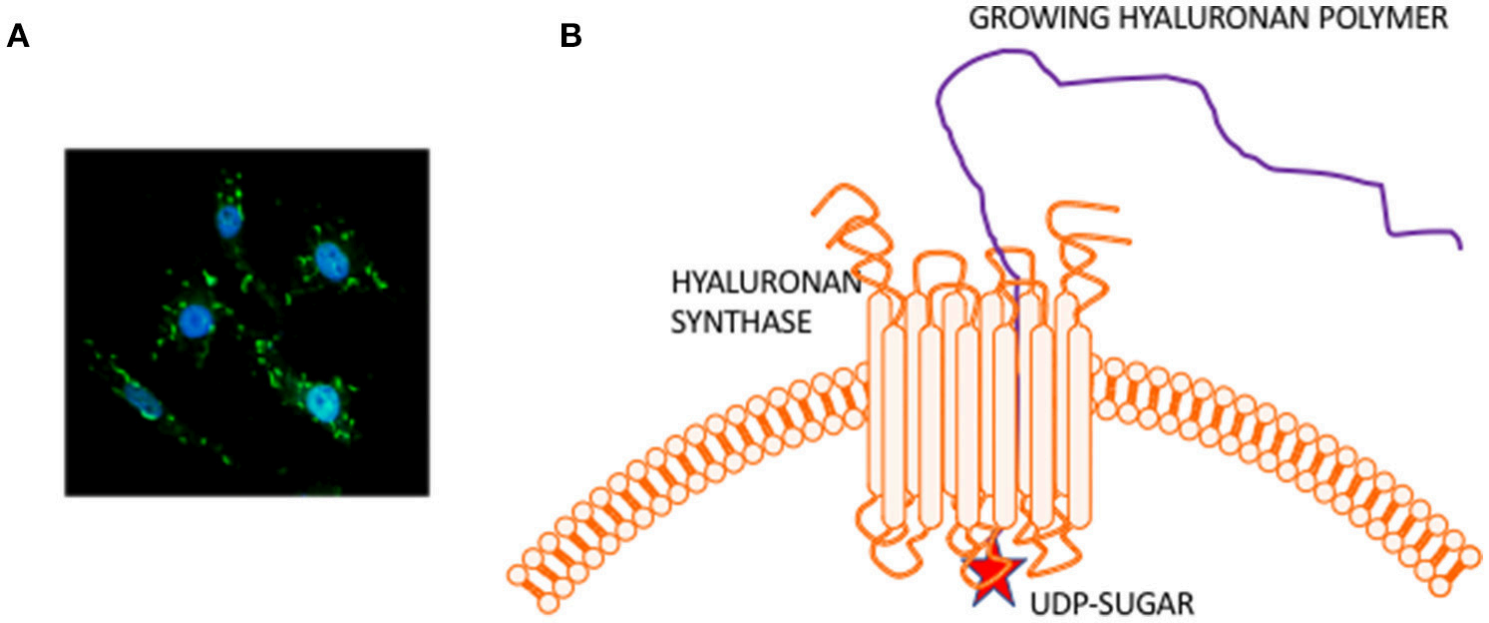
Hyaluronan is a simple polysaccharide produced by cell membrane synthases. **(A)** Micrograph showing membrane localized hyaluronan synthase 2 in fibroblasts. **(B)** Hyaluronan synthase has multiple transmembrane domains that cluster to form pores in the cell membrane. UDP-sugars bind to the protein cytoplasmic face and growing polymer is extruded through the pore to the extracellular space.

**Figure 3:**
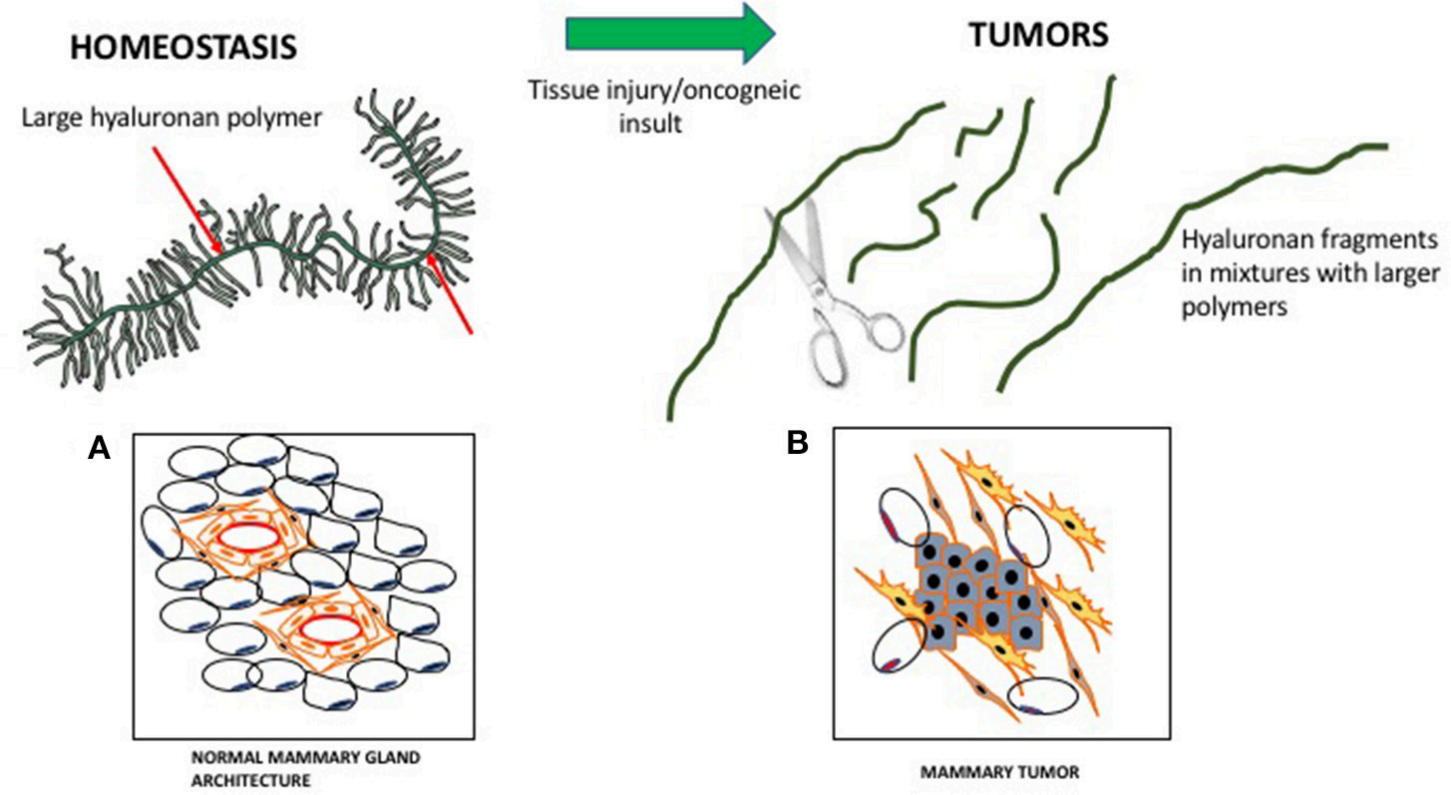
Hyaluronan is a large polymer during tissue homeostasis and fragmented in wounds and tumors. In homeostasis, large hyaluronan polymers are decorated by proteins/proteoglycans, which contribute to normal tissue architecture. **(A)** Cartoon of a virgin mouse mammary gland. **(B)** Cartoon of a mouse mammary tumor (Blue, mammary tumor cells). During wounding, fragmented hyaluronan polymers produced by ROS/hyaluronidases activate fibroblasts and attract immune cells, which contribute to the loss of tissue architecture.

HA is composed of repeating disaccharide units of N-acetylglucosamine and ß-glucuronic acid linked together by three highly homologous synthases (HAS1,2,3). These are most frequently located at the plasma membrane and the growing HA polymer is extruded directly into the extracellular space through pores in the plasma membrane formed by synthase oligomerization (Weigel, 2015) (e.g., Figure 2). Evolving evidence indicates that the biological effects of HA are primarily determined by size rather than conformational changes typically required for protein activation. In general, large HA polymers, which are mainly present in homeostatic tissues, are immunologically quiescent and contribute to enforcing cell survival and homeostasis. HA fragments (e.g., < 100–200 kDa), which are generated by reactive oxygen/nitrogen species (ROS/RNS) and hyaluronidases produced during tissue stress, repair and chronic disease, are pro-inflammatory and pro-fibrotic (Simpson and Lokeshwar, 2008; Gaudet and Popovich, 2014; Cyphert et al., 2015; Sherman et al., 2015; Gaggar and Weathington, 2016; Maytin, 2016; Turley et al., 2016; Bohaumilitzky et al., 2017; Cowman, 2017; Frevert et al., 2017; Kavasi et al., 2017; Wight et al., 2017; Wu et al., 2017; Avenoso et al., 2018a,b) (Figure 3). The precise effect of specific sizes of HA fragments on immune and mesenchymal cells on such functions as gene expression appears to be cell-context and stimulus-specific, and is currently controversial (Cowman, 2017; Weigel, 2017; Weigel and Baggenstoss, 2017). HA fragment accumulation in quiescent homeostatic tissues is low. In contrast remodeling and diseased tissues such as cancers often contain an elevated level of HA (e.g., Teder et al., 2002; Koyama et al., 2007; Li et al., 2011; Tolg et al., 2017), clear evidence of fragmentation, and overexpression of HAS, hyaluronidases and HA receptors.

The tumor-resistance properties of high molecular weight HA were originally identified in the tumor resistant naked mole rat and resistance of fibroblasts to oncogenic transformation was shown to depend upon production of large HA polymers (Tian et al., 2013). Naked mole rat tissues contain larger HA polymers and less detectable fragmentation than tissues of the more tumor-susceptible mouse. HA-mediated tumor resistance of the naked mole rat is attributed to the ability of high molecular weight HA to hyper-sensitize cells to contact inhibition and induce p16 (ink4a) locus expression with consequent cell cycle arrest (Tian et al., 2015). Consistent with this explanation, HA overproduction has also been shown by other groups to regulate contact inhibition and adhesion in cultured non-malignant cells (Itano et al., 2008). Others have shown that excess production of HA by itself does not promote an aggressive tumor phenotype and can even be tumor-suppressing by blocking G1-S transition in the cell cycle (Bharadwaj et al., 2011). Similarly, exposure of tumor cells to hyaluronidases alone (e.g., HYAL1 or PH-20) can be growth-suppressing (Simpson and Lokeshwar, 2008) and increase response of tumor cells to therapy (Wong et al., 2017). Thus, high HA production combined with an increased capacity for polymer fragmentation appears to be responsible for oncogenic effects of this polysaccharide.

A number of studies using mouse models also predict that elevated HA production, primarily by fibroblasts, pre-disposes epithelial cells to tumor initiation. Examples include evidence that an HA-rich stroma precedes increased mammary tumor formation in transgenic mice expressing both MMTV-driven HAS2 and a c-neu proto-oncogene. HAS2/c-neu mice tumors notably produce higher levels of both high molecular weight and fragmented HA than the c-neu mice (Koyama et al., 2007). Using p38MAPK knock-in mice and tumor xenografts, others have shown that MAPK-driven HAS2 expression and consequent HA production by fibroblasts is required for their activation into CAFs and for loss of their tumor suppressing properties resulting in a pro-tumor niche and increased lung colonization (Brichkina et al., 2016). These studies suggest that the tumor suppressing effects of either HA or processing enzymes alone are converted into a pro-tumor stimulus when HA processing into fragments is enhanced and sustained by elevated expression of one or more HAS genes, and hyaluronidases, often HYAL1. Additional studies predict that the pro-tumor functions of HA also depend upon the display of specific receptors, notably the injury-related HA receptor, RHAMM (gene name HMMR), which activates oncogenic signaling pathways (Tolg et al., 2014; Misra et al., 2015; Nikitovic et al., 2015; Schwertfeger et al., 2015). In a pre-malignant stroma, these genes are expressed by CAF-like fibroblasts.

## CAFs and tumor dissemnation

CAFs play a significant role in tumor dissemination by inducing an invasive phenotype in tumor cells, promoting motile phenotypes and remodeling the ECM. Invasion is achieved in part by CAF-driven EMT and consequent cell migration driven by factors such as TGF-B, HGF, and CXCL12/SDF-1(Kalluri, 2016). Paladin-expressing CAF create “tunnels” in the ECM which cancer cells migrate through (Brentnall, 2012). Under CAF guidance, tumor cells also migrate and invade as groups in the absence of apparent EMT. This collective migration and invasion is driven by heterotypic E-cadherin/N-cadherin interactions between tumor cells and CAFs (Labernadie et al., 2017) that results in a mechanically active adhesion. CAF-mediated ECM remodeling occurs as a result of secretion of collagen, proteases, and in particular, matrix metalloproteinases. ECM remodeling provides a microenvironment that further supports tumor cell migration and dissemination. Interestingly, CAFs from different breast cancer molecular subtypes including Luminal A, Her2-like, and triple negative/basal-like exhibit subtype-specific differences in stromal gene expression (Tchou et al., 2012), microRNA expression and secretory profiles (Shah et al., 2015). Furthermore, CAFs from more aggressive cancers induce more aggressive breast cancer cell phenotypes than CAFs from more indolent cancers (Shah et al., 2015).

**Figure 4:**
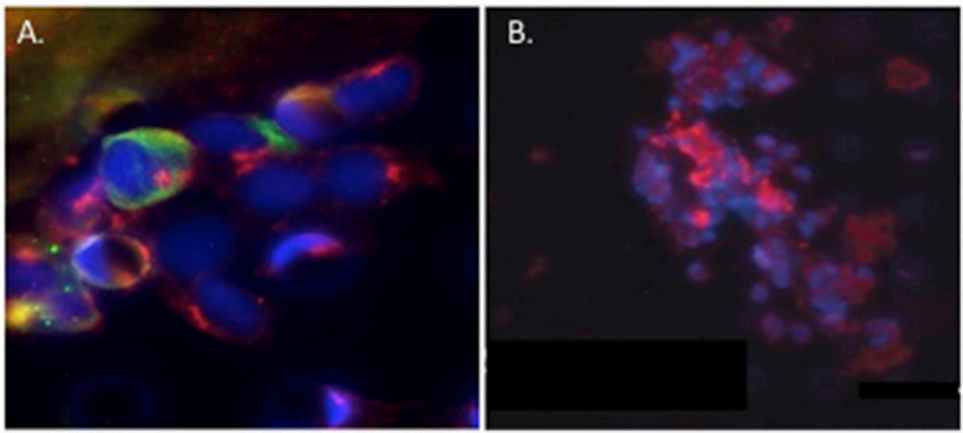
Circulating cCAF/circulating tumor cell (CTC) clusters and cCAF clusters in breast cancer patient blood. **(A)** cCAF/CTC co-cluster and **(B)** cCAF cluster. Red: FAP, Green: CK. From Ao et al. (2015).

Circulating tumor cell (CTC) clusters were originally described in the 1970's and are now considered to be pre-cursors of metastatic colonies. In mouse breast cancer models, circulating tumor cell clusters exhibit higher metastatic capacity compared with individual or single CTCs (Aceto et al., 2014). Additionally, polyclonal breast cancer metastases have been suggested to arise from circulating tumor cell clusters composed of Keratin 14+ cells (Cheung et al., 2016). Quantification of these CTC clusters in breast cancer patients show that their presence correlates with reduced progression-free survival and poor outcome (Cheung et al., 2016; Jansson et al., 2016; Mu et al., 2016; Wang et al., 2017). Collective migration of tumor cell clusters into the circulation appears to offer a tumor cell survival advantage compared to entry of single tumor cells into the vasculature. CAFs are not only present in primary and metastatic tumor stroma but have recently been shown to occur in the circulation either as individual CAFs, part of CTC clusters or as CAF clusters. Circulating CAFs (cCAFs) likely contribute to CAFs found in pre-metastatic and metastatic niches. Mouse metastasis models suggest that circulating CAFs can exit either with groups of cancer cells or by themselves. In these models, the presence of CAFs from the primary TME promotes metastatic seeding and growth (Duda et al., 2010), likely by helping to create a suitable growth and survival microenvironmental niche for tumor cells and to aid in avoidance of immune surveillance. Additionally, since CAFs are present in pre-metastatic niches prior to the appearance of tumor cells, circulating CAFs likely also play a role in establishing or preparing a niche suitable for future tumor cell colonization. In a pilot study, cCAFs were detected in the blood from patient with Stage IV (metastatic) breast cancer but not from patients with Stage I disease with no evidence of relapse, while CTCs were detected in both patient samples (Ao et al., 2015). Furthermore, CTCs and cCAFs circulate in co-clusters in patient blood, and like CTCs, cCAFs can also cluster with each other (Figure 4). Jones and colleagues also found circulating CK-/CD45/VIM+ fibroblast-like cells in metastatic prostate cancer patient blood (Jones et al., 2013). The development of techniques for isolating circulating CAFs from mouse models of human breast cancer xenografts and mammary tumor susceptibility will greatly aid in characterizing both the origin and contribution of circulating CAFs to successful metastasis. Recent evidence suggests that at least a portion of CTCs are tumor cells transitioning between the epithelial and mesenchymal state (Yu et al., 2013) that possess stem cell-like properties and phenotypically plasticity May et al., 2011. Functional characterization of these circulating cells/clusters will clarify the mechanisms of tumor cell dissemination and likely identify potential therapeutic targets for metastatic disease.

## Hyaluronan and tumor dissemination

**Figure 5:**
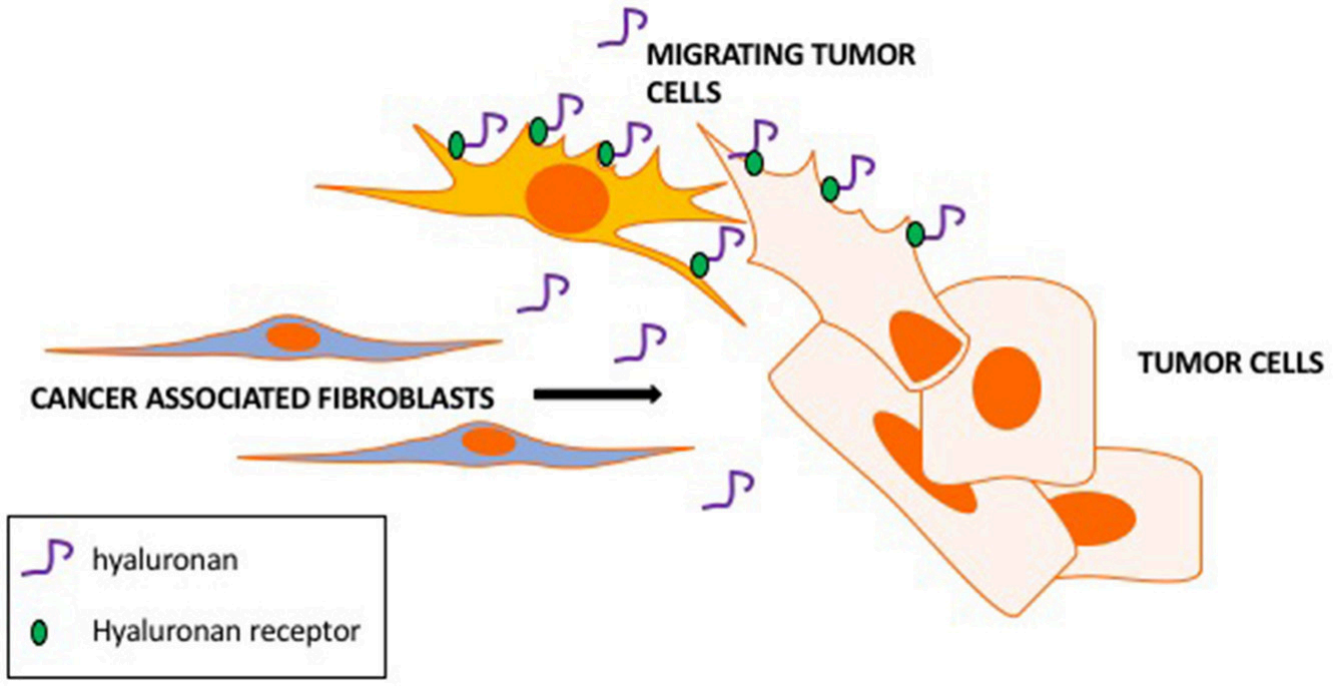
Hyaluronan promotes CAFs motility toward tumor cells and tumor cell motility. CAF subsets produce hyaluronan as a motogenic stimulus for migrating toward tumor cells. Hyaluronan binds to tumor cell subsets via hyaluronan receptors (RHAMM and CD44) contributing to the migration and invasion of CAF-guided tumor cells.

A CAF property that appears to be critical to cancer cell invasion is their active motility and tropism toward tumor cells (e.g., Costea et al., 2013; Berdiel-Acer et al., 2014). These properties culminate in close physical heterotypic contact (Marusyk et al., 2016; Labernadie et al., 2017). Clinically, close proximity of CAFs to tumor cells is linked to poor outcome and resistance to therapy and supports migration and invasion of tumor cells by several mechanisms (Marusyk et al., 2016). HA is one CAF-produced ECM factor that appears to play a key role in these critical autocrine and paracrine migratory interactions of CAFs and tumor cells. Thus, highly motile CAF subtypes produce and rely upon HA for their motogenic properties (Costea et al., 2013) and ability to promote migration of tumor cells. We and others (e.g., Hamilton et al., 2007; Mele et al., 2017; Shigeeda et al., 2017) have also reported that highly aggressive breast cancer cells that have undergone EMT develop a CAF-like autocrine production of HA to sustain their high motility rates. Such tumor cells are able to invade independently from CAFs (Turley et al., 2016). Intriguingly, we have shown using fluorescent HA-probes that HA-binding to breast cancer cells and to activated fibroblasts is heterogeneous (Veiseh et al., 2014, 2015). FACS-sorted tumor cell subsets that bind high levels of HA are more motile, invasive and metastatic than subsets that bind low or no probe. A concept that emerges from these studies is that CAF subsets not only utilize HA to migrate close to tumor cells but that their autocrine production of HA also stimulates the migration of the HA binding tumor subpopulation (Figure 5). Expression of HA receptors CD44 and RHAMM is required for migration of these tumor cells, and we predict that these receptors also mediate HA-dependent, highly motile CAF subsets.

The role of HA and its receptors in circulating CAFs and tumor cells is currently understudied. However, several studies have reported that circulating tumor cells from cancer patients express the HA receptor CD44 (Grillet et al., 2017) and can be captured from circulation by adhering to HA, a process that is mediated by HA receptors (Xu et al., 2017). Interestingly, circulating cells with this dual phenotype are EpCAM- and are therefore distinct form the more commonly studied EpCAM+ circulating tumor cells (Mirza et al., 2017). EpCAM-/CD44+ cells may represent tumor cells that have undergone EMT and/or are circulating cancer stem cells (cCSCs). Circulating cells isolated from lung adenocarcinoma patients that had higher levels of markers such as RHAMM (HMMR) had shorter survival times (Man et al., 2014). CAFs also express CD44 and this CD44 plays important roles in CAF function. These collective results predict a critical importance of HA production and HA receptor display in cCAFs and CTCs to successful metastases.

## Targeting stroma and CAFs

Targeting key genetic or epigenetic alterations in tumors and/or the use of immune checkpoint inhibitors has significantly improved cancer therapy (Jiang et al., 2017). While these advances are encouraging, they are currently either effective in a minority of cancer patients, have significant pro-tumor side-effects or lack long-term durability. Thus, new approaches are necessary to expand the number of patients who will benefit clinically from chemotherapy and targeted therapy. Targeting the fibrotic stroma is emerging as a potentially key approach necessary to achieving therapeutic efficacy. This is particularly true for pancreatic cancer, which typically progresses with an extensive fibrotic stroma that can account for over 80% of the tumor volume (Yu and Tannock, 2012; Tan et al., 2015). Therapies that target the fibrotic stroma, including HA, are being developed and entering clinical trials (Provenzano and Hingorani, 2013; Jiang et al., 2017; Kumari et al., 2017).

High interstitial pressures in the fibrotic stroma of pancreatic cancers, which results from high production of collagen and HA, causes the collapse of the stromal vasculature in pancreatic cancers and impedes exposure of tumor cells to chemo- and immune therapies (Yu and Tannock, 2012). Multiple approaches to target fibrotic stroma are therefore being tested to overcome these delivery issues. One successful strategy is targeting HA. Systemic administration of a recombinant sperm hyaluronidase (PEGPH20), degrades hyaluronan in pancreatic cancer stroma (Provenzano and Hingorani, 2013). This destruction decreases interstitial fluid pressure, increases vasculature patency and improves the delivery of gemcitabine. Importantly, these hyaluronidase-mediated changes both decrease tumor volume and increase animal survival in experimental models of pancreatic cancer. PEGPH20 is now in phase III clinical trials for pancreatic cancer (Doherty et al., 2018). An alternative to the use of recombinant hyaluronidase has been of HA synthesis inhibitors (e.g., 4-methylumbelliferone), which also inhibits tumor growth and could be used in alone or in combination with hyaluronidase to improve therapeutic response (Kudo et al., 2017).

CAF-targeted therapies are also being developed to blunt their fibrosis-activated signaling. For example, a selective FAK inhibitor (VS-4718) targets hyperactive FAK in stromal CAFs. This inhibitor reduces fibrosis, decreases the number of tumor-infiltrating immuno-suppressive cells and results in survival doubling in mouse models of pancreatic ductal adenocarcinoma (Jiang et al., 2016, 2017). Inhibiting FAK activation also increases responsiveness to chemotherapy and immune checkpoint inhibitors with resulting improved outcome. These pre-clinical successes have led to phase 1 clinical trials using this FAK inhibitor in combination with immune checkpoint inhibitors (Jiang et al., 2017). While FAK hyper-activation is a key feature of mechano-signaling in CAFs and provides a proof-of-concept for targeting the microenvironment, stromal immune cells also utilize FAK or the related PYK-2 for survival (Jiang et al., 2017). Off target effects of VS-4718 could contribute to immune-suppression and therefore compromise its effective utility in humans (Jiang et al., 2016).

Active investigations are also underway to target CAF survival in the fibrotic stroma. In contrast to carcinoma cells, CAFs are genetically normal cells that have been co-opted and modified by cancer cells into a state of constitutive activation. CAFs therefore have a less plastic genome than tumor cells limiting their ability to rapidly modify their genome but making them an attractive candidate for stable responses to targeted therapy. CAFs uniquely express FAP, which plays important roles in CAF function (Lai et al., 2012; Koczorowska et al., 2016). *In vivo* administration of a FAP enzyme inhibitor, Talabostat, in tumor-bearing mice results in tumor regression and upregulation of specific chemokines and cytokines that induce an anti-tumor immune response (Cunningham, 2007). Talabostat is well tolerated in healthy volunteers in both Phase I and II clinical trials but does not result in therapeutic benefit. A CAF-directed, anti-human FAP antibody, sibrotuzumab (Fischer et al., 2012), exhibits specificity and activity in preclinical mouse models (Fischer et al., 2012), and was well tolerated in early Phase I/II clinical trials (Hofheinz et al., 2003; Scott et al., 2003) but has failed to show therapeutic activity in patients with metastatic disease. FAP-targeted chimeric antigen receptor (CAR) T cells reduce ECM, vessel density, and growth of several types of human cancer xenografts and murine pancreatic cancers when introduced into mice by adoptive transfer (Wang et al., 2014; Lo et al., 2015). This technology has not yet entered clinical trials. FAP may be useful for targeting therapies to CAFs. Potentially the development of therapies that impede CAF survival/function in the circulation or their ability to migrate/enter the circulation (e.g., HA/RHAMM) may be a more promising approach.

In conclusion, despite recent advances in targeted therapies, metastases, recurrence and relapse remain as major clinical obstacles to successful cancer treatment. Carcinoma cell epigenetic and genetic heterogeneity are important factors that limit therapeutic efficacy. However, a wealth of studies has now demonstrated that tumor-associated fibrotic stroma is also a major contributing factor to therapeutic failure. The success of new approaches to targeting tumor cells will in the future likely have to include agents that compromise the pro-tumorigenic fibrotic ECM.

## Author contributions

ET: Organized, referenced, edited contributions and wrote introduction, and sections on CAF-mediated tumor initiation, relationship of microenvironment and hyaluronan, prepared model Figures 1, 2, 3. JM: Edited contributions, wrote abstract and wrote/referenced sections on tumor dissembination, biomechanical properties of tumor stroma and therapeutic approaches to targeting CAFs and fibrotic stroma. DE-A: Edited contributions wrote/referenced section on circulating tumor cells and CAFs, tumor dissemination and prepared Figure 4.

### Conflict of interest statement

The authors declare that the research was conducted in the absence of any commercial or financial relationships that could be construed as a potential conflict of interest.
