# Corrigendum: Hyaluronan, Cancer-Associated Fibroblasts and the Tumor Microenvironment in Malignant Progression

**DOI:** 10.3389/fcell.2018.00112

**Published:** 2018-09-24

**Authors:** James B. McCarthy, Dorraya El-Ashry, Eva A. Turley

**Affiliations:** ^1^Department of Laboratory Medicine and Pathology, Masonic Comprehensive Cancer Center, Minneapolis, MN, United States; ^2^London Regional Cancer Program, Department of Oncology, Biochemistry and Surgery, Schulich School of Medicine and Dentistry, Lawson Health Research Institute, Western University, London, ON, Canada

**Keywords:** hyaluronan, cancer-associated fibroblasts, migration, tumor Microenvironment, tumor initiation, circulating cancer-associated fibroblasts, metastasis

In the original article, there was a mistake in Figure 4 as published. We erroneously included a figure of unpublished data that should not have been included. A new figure demonstrating the same key point of heterotypic co-clusters of CTCs and cCAFs with corrected text describing the figure (see below), as well as a new figure legend is being provided. The corrected [Figure 4] appears below and a correction has been made to CAFS AND TUMOR DISSEMINATION, Paragraph Number 2:

**Figure 4:**
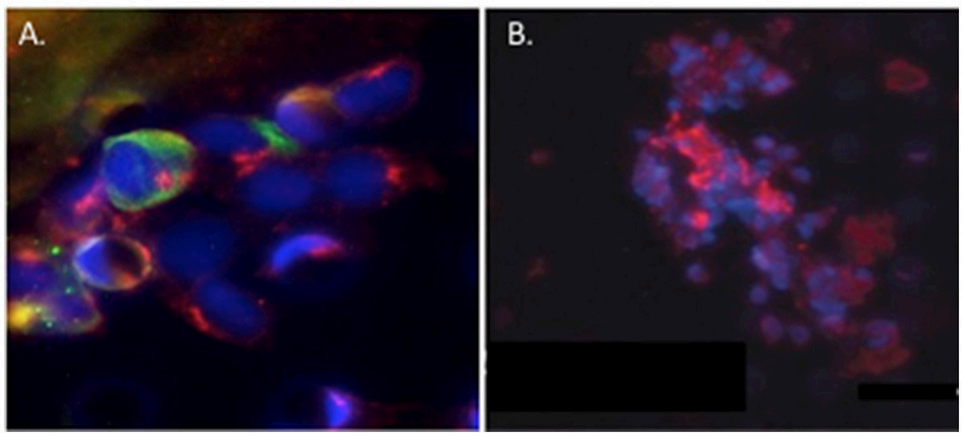
Circulating cCAF/circulating tumor cell (CTC) clusters and cCAF clusters in breast cancer patient blood. **(A)** cCAF/CTC co-cluster and **(B)** cCAF cluster. Red: FAP, Green: CK. From Ao et al. (2015).

Circulating tumor cell (CTC) clusters were originally described in the 1970's and are now considered to be pre-cursors of metastatic colonies. In mouse breast cancer models, circulating tumor cell clusters exhibit higher metastatic capacity compared with individual or single CTCs (Aceto et al., 2014). Additionally, polyclonal breast cancer metastases have been suggested to arise from circulating tumor cell clusters composed of Keratin 14+ cells (Cheung et al., 2016). Quantification of these CTC clusters in breast cancer patients show that their presence correlates with reduced progression-free survival and poor outcome (Cheung et al., 2016; Jansson et al., 2016; Mu et al., 2016; Wang et al., 2017). Collective migration of tumor cell clusters into the circulation appears to offer a tumor cell survival advantage compared to entry of single tumor cells into the vasculature. CAFs are not only present in primary and metastatic tumor stroma but have recently been shown to occur in the circulation either as individual CAFs, part of CTC clusters or as CAF clusters. Circulating CAFs (cCAFs) likely contribute to CAFs found in pre-metastatic and metastatic niches. Mouse metastasis models suggest that circulating CAFs can exit either with groups of cancer cells or by themselves. In these models, the presence of CAFs from the primary TME promotes metastatic seeding and growth (Duda et al., 2010), likely by helping to create a suitable growth and survival microenvironmental niche for tumor cells and to aid in avoidance of immune surveillance. Additionally, since CAFs are present in pre-metastatic niches prior to the appearance of tumor cells, circulating CAFs likely also play a role in establishing or preparing a niche suitable for future tumor cell colonization. In a pilot study, cCAFs were detected in the blood from patient with Stage IV (metastatic) breast cancer but not from patients with Stage I disease with no evidence of relapse, while CTCs were detected in both patient samples (Ao et al., 2015). Furthermore, CTCs and cCAFs circulate in co-clusters in patient blood, and like CTCs, cCAFs can also cluster with each other (Figure 4). Jones and colleagues also found circulating CK-/CD45/VIM+ fibroblast-like cells in metastatic prostate cancer patient blood (Jones et al., 2013). The development of techniques for isolating circulating CAFs from mouse models of human breast cancer xenografts and mammary tumor susceptibility will greatly aid in characterizing both the origin and contribution of circulating CAFs to successful metastasis. Recent evidence suggests that at least a portion of CTCs are tumor cells transitioning between the epithelial and mesenchymal state (Yu et al., 2013) that possess stem cell-like properties and phenotypically plasticity (May et al., 2011). Functional characterization of these circulating cells/clusters will clarify the mechanisms of tumor cell dissemination and likely identify potential therapeutic targets for metastatic disease.

In addition, there was an error in the Author Contributions Statement. It was stated that ET prepared the model for Figure 4 and DE-A for Figure 3 when it was in fact the other way around. ET prepared the model for Figure 3 and DE-A for Figure 4.

The authors apologize for this error and state that this does not change the scientific conclusions of the article in any way.

The original article has been updated.

## Conflict of interest statement

The authors declare that the research was conducted in the absence of any commercial or financial relationships that could be construed as a potential conflict of interest.
